# PD220 Clinical Expert Opinion To Inform Health Technology Assessment In Ireland

**DOI:** 10.1017/S0266462324004392

**Published:** 2025-01-07

**Authors:** Joan O’Callaghan, Lesley Tilson, Emer Fogarty, Laura McCullagh

## Abstract

**Introduction:**

The National Centre for Pharmacoeconomics (NCPE) in Ireland conducts health technology assessments (HTAs) of drugs under consideration by the decision-maker for reimbursement. We analyzed how clinical expert opinion obtained by applicant pharmaceutical companies is used to inform HTA submissions made to the NCPE. We also describe how clinical opinion obtained by the NCPE is used to inform NCPE assessments.

**Methods:**

We conducted a retrospective review of HTA submissions made to the NCPE from July 2019 to June 2020 inclusive. Data were extracted using a bespoke data collection instrument created in Microsoft Excel. To describe how clinical opinion informed the NCPE assessments, we extracted data from NCPE HTA Technical Summary Reports available on the NCPE website.

**Results:**

A total of 18 HTA submissions were reviewed. Clinical expert opinion was used by applicants to support all submissions. The median number of clinical experts who informed each individual HTA submission was seven (range 1 to 33); the majority were hospital physicians. Clinical opinion was used to inform HTA domains, including patient and population estimates (n=14; 78%), use of drugs in clinical practice (n=13; 78%), treatment effectiveness (n=6; 33%), healthcare resource use (n=14; 78%), and health-related quality of life (n=5; 28%). We present examples where clinical opinion, obtained by the NCPE, was used to inform NCPE assessments.

**Conclusions:**

Clinical expert opinion informed all 18 applicant HTA submissions made to the NCPE during the study period. The NCPE also seeks clinical expert opinion to inform their assessments. Healthcare professionals make an important contribution to HTA and, thus, inform the decision-making processes on drug reimbursement in Ireland.

